# Dental Works in Progress

**Published:** 1841-12

**Authors:** 


*Dental Works in Progress.*—We understand that Dr. W. N. McKinney, surgeon dentist of Fredericksburg, Va. is preparing a popular treatise on the management of the teeth, and that it will soon be put to press. Dr. McKinney is a very ready writer, and knowing which, we hope that it will not be long before his promised work makes its appearance.

We have also been informed, that Dr. Pleasants, dentist, of Richmond, Va. is engaged in the preparation of a work on the science of the teeth, and that he contemplates very soon, to have it published.

